# Increased Attentional Focus Modulates Eye Movements in a Mixed Antisaccade Task for Younger and Older Adults

**DOI:** 10.1371/journal.pone.0061566

**Published:** 2013-04-19

**Authors:** Jingxin Wang, Jing Tian, Rong Wang, Valerie Benson

**Affiliations:** 1 Academy of Psychology and Behaviour, Tianjin Normal University, Tianjin, China; 2 Department of Humanities and Social Science, Taiyuan University of Science and Technology, Taiyuan, China; 3 Centre for Visual Cognition, School of Psychology, University of Southampton, Southampton, United Kingdom; University of Leicester, United Kingdom

## Abstract

We examined performance in the antisaccade task for younger and older adults by comparing latencies and errors in what we defined as high attentional focus (mixed antisaccades and prosaccades in the same block) and low attentional focus (antisaccades and prosaccades in separate blocks) conditions. Shorter saccade latencies for correctly executed eye movements were observed for both groups in mixed, compared to blocked, antisaccade tasks, but antisaccade error rates were higher for older participants across both conditions. The results are discussed in relation to the inhibitory hypothesis, the goal neglect theory and attentional control theory.

## Introduction

The antisaccade task, devised by Hallett in 1978 has been used extensively over the past 30 years to investigate inhibition and voluntary control in both normal and clinical populations [1]–[6]. Recently the task has been adopted to examine cognitive processing deficits in ageing populations [7]–[11]. This is because an important cognitive function that has been shown to decline in this population is the ability to inhibit inappropriate prepotent responses for a range of tasks [12]–[13]. Prepotent responses may be described as habitual or reflexive responses. For the purpose of this study a prepotent response is defined as an eye movement executed to the target, rather than to the opposite location of the target.

In antisaccade experiments participants are required to make an eye movement (saccade) either toward (prosaccade) or away from (antisaccade) a peripherally presented target. Although prosaccades are often described as being executed reflexively [14], it is acknowledged that they have a voluntary component to them and that they are at least 100 ms longer than actual reflexive (entirely stimulus driven) saccades [15]. Correct performance on antisaccade trials requires the inhibition of a prepotent reflexive eye movement towards the target, and the generation and execution of a correct saccade in the opposite direction. Antisaccades are therefore essentially voluntary saccades, generated via top-down cognitive control (goal driven). Compared to prosaccade trials, the top down volitional control required for antisaccade trials results in increases in the time taken (latency) to initiate correct saccades in the opposite direction to the target. When top-down control fails in the antisaccade trials this results in a directional error, where the eye moves towards the target, rather than in the opposite direction. Errors are stimulus, rather than goal driven, and reflect bottom-up processing.

Older adults perform poorly in the antisaccade task where they exhibit higher rates of erroneous saccades directed to the target and also show prolonged saccade latencies for antisaccades [7]–[11], [13], [16]–[17]. This deficit in performance has been attributed to either the failure to “inhibit” the prepotent response to the visual stimulus [7], [12], [18], or to increasing “goal neglect” [19]–[21].

The inhibitory deficit hypothesis of ageing proposes that age-related changes in the antisaccade task result from a reduced ability to inhibit making an eye movement towards the target when such a prepotent response would be incorrect [22]–[23]. An alternative explanation for poorer performance in older adults is the goal neglect theory [19]. Goal neglect has been broadly defined as “the disregard of a task requirement even if it has been understood”, thus, deficits in performance are due to a failure to maintain the current task goal, resulting in goal neglect or goal decay [24]. Whilst it has been suggested that goal neglect and inhibition are two separate processes they may not however be mutually exclusive. For example, it has been proposed that goal activation is simply the inverse of inhibition [10]. This description though is rather narrow, and does not take account of the view that whilst maintaining the current goal could result in suppression of distractor related activity during antisaccade trials, goal activation would also facilitate responding to goal relevant information.

An important factor that could affect performance in the antisaccade task in older adults is the role of attention, in both goal maintenance of the task and inhibition of prepotent responses. It has recently been shown that attentional cueing can modulate performance in older adults in some tasks [25]. Using a visual search paradigm where distractors had to be ignored, it was found that having to pay more attention (allocate more attentional resources to one side of space) throughout the task increased the ability to be less distracted by the presence of task irrelevant visual distractors appearing elsewhere in the display, which resulted in reduced proportions of errors in older participants. Having to pay more attention in an antisaccade task could potentially improve performance in older adults if that results in an increased ability to ignore the distractor in antisaccade trials.

People have argued that the role of executive attention would be crucial in the antisaccade task, because of the need to actively maintain the task goal in the likelihood of a powerful attention-capturing cue resulting in a habitual response opposite to the one required in the task [26]. Executive attention includes, amongst other things, planning actions and allocating attention to goals. Of particular relevance to the antisaccade task is the capacity of working memory for the maintenance of the current task goal [27]. Increasing the attentional demands on working memory has been shown to result in poorer cognitive performance, such as increases in correct saccade latencies and in the proportion of directional errors for a group of younger participants with low working memory capacity in an antisaccade task [24].

Thus, for a range of tasks, attention has been shown to influence performance in both younger and older adults. The aim of this experiment was to investigate whether increasing the level of attentional focus to be maintained whilst completing an antisaccade task modulated either the proportion of errors and/or the latencies of the correct responses for younger and older participants. To achieve this we adopted a mixed antisaccade design whereby antisaccades are presented randomly with prosaccades in blocks of trials, or where each type of saccade is presented in isolation in a single block of trials. It is assumed that the attentional focus is increased in the mixed, compared to the blocked task because the type of eye movement that participants have to execute (prosaccade or antisaccade) is signaled by a cue that must be attended to on every trial [28]. Attending to the cue on each trial, we suggest, increases the focus of attention needed for mixed antisaccade blocks of trials, compared to a condition where the type of saccade remains constant throughout the block. A principal question of interest was whether having to pay attention to the cue on every trial in that condition would be reflected in any differences in performance for mixed versus blocked antisaccade tasks and whether such differences could tell us anything new about how deficits in older participants relate to either difficulties in maintaining the goal of the experimental task (goal neglect), or an inability to suppress a prepotent response (inhibition).

According to the inhibition theory, poorer performance in older participants should result in the error rates for antisaccade trials being greater for the older participants regardless of whether the trials are blocked or mixed, because inhibition is assumed to be unaffected by any differences in goal activation. Older participants will also show longer latencies in the antisaccade task compared to the younger participants, because it will take them longer to inhibit a prepotent response, but again this should be observed across conditions.

According to the goal neglect theory, correct performance in conflict situations is dependent on adequate activation, or availability, of current task goals. If goal activation is insufficient when the stimulus is presented during the task a prepotent response, an eye movement towards the target, will be executed resulting in a greater proportion of errors in this condition. We propose that, as a result of an increase in the focus of attention in the mixed condition goal activation should be higher in that condition because participants have to attend to the cue on every trial in order to make a saccade of the correct type. Therefore, if poor goal neglect is what is driving decreased performance in older people in previously reported antisaccade studies, we would expect to find a decrease in error rates and latencies for antisaccades in a mixed compared to a blocked design, for older participants.

It should be noted that previous research examining blocked versus mixed conditions in the antisaccade paradigm has yielded inconsistent results to date. Some studies have shown that there are either no differences in latencies or errors across the two conditions, whereas others have reported that performance actually decreases in the mixed condition compared to the blocked condition [29]–[30]. Nevertheless, there is also evidence of better performance in a mixed rather than a blocked antisaccade task [28]. This is known as the paradoxical effect of a switch benefit on latencies but not on errors. We will therefore also examine whether there was any switch cost or benefit in this study, and whether these effects were related to the participant age group.

## Methods

### Ethics Statement

This study was approved by the Institutional Review Board of Tianjin Normal University, and every participant provided written informed consent before taking part in the experiment.

### Participants

Because practice affects performance in the antisaccade task, two different groups of participants completed each task [31]–[32]. Participants in both tasks had no known neurological, psychiatric, or visual disorders, and were paid for their participation. The two groups were matched on years of education (younger 15.1, older 14.8), and the Wechsler Adult Intelligence Scale vocabulary subset (younger 60.83 (range from 53 to 80), older 58.72 (range from 47 to 75), *t*(78) = 1.05, *p*>.05) [33]. Younger participants were volunteers from Tianjin Normal University (China), and older volunteers were recruited from the Tianjin College for the elderly in Tianjin Normal University (China).

Blocked condition: Participants were 20 younger adults (mean age = 22.7 years old (range from 19 to 27), 6 male and 14 female) and 20 older adults (mean age = 63.0 years old (range from 55 to 77), 8 male and 12 female).

Mixed condition: Participants were 20 younger adults (mean age = 22.9 years old (range from 20 to 27), 6 male and 14 female) and 20 older adults (mean age = 64.1 years old (range from 54 to 77), 8 male and 12 female).

There was no significant difference between the older adults in the two conditions in terms of participant age (*t*(38) = −0.29, *p*>.77), or the younger adults in the two conditions (*t*(38) = −0.45, *p*>.65).

### Apparatus and Materials

We recorded eye movements of the right eye using an EyeLink eye-tracking system (SR Research Ltd.) with a sampling frequency of 500 Hz. Participants were seated in a comfortable chair and the distance was 75 cm away from a 19-in monitor with a resolution of 1024×768 pixels. A chin rest stabilized head position.

**Figure 1:**
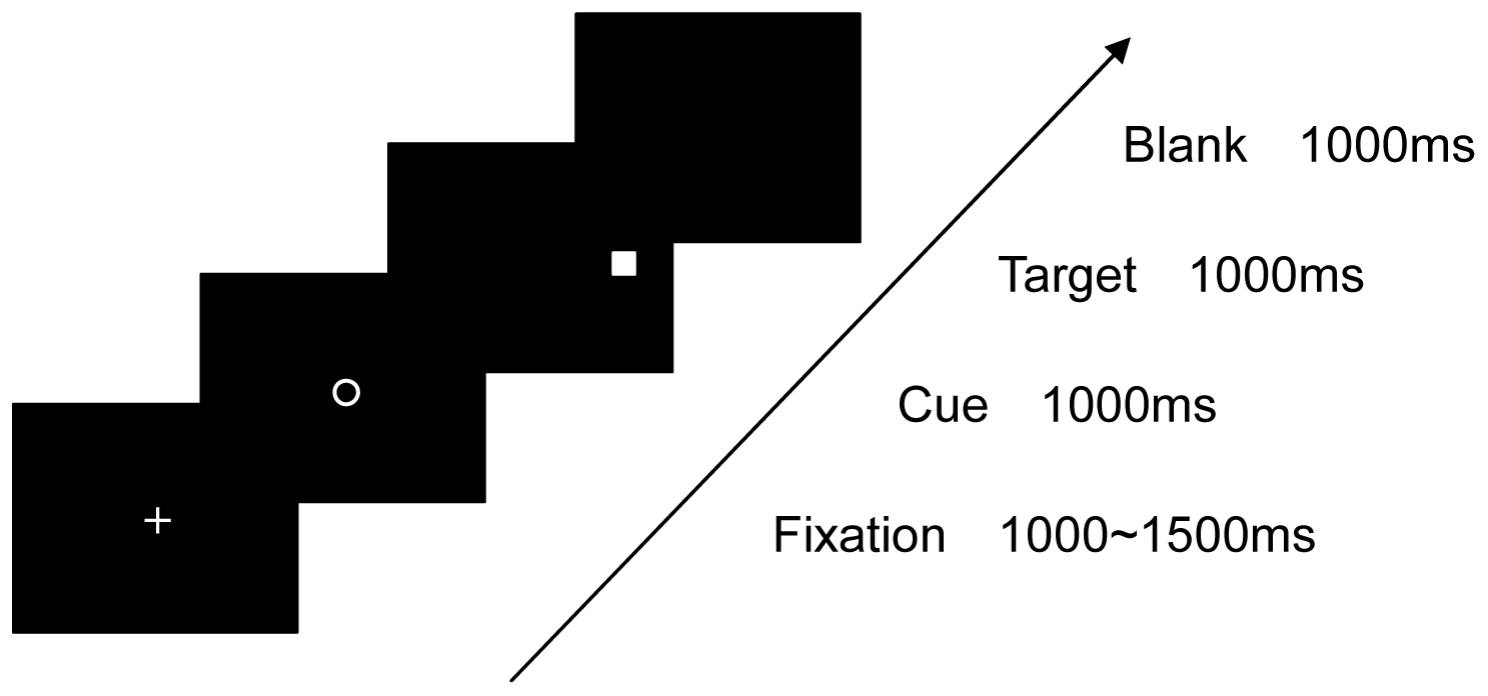
A schematic of the trial sequence for both experimental conditions. In the blocked condition the symbolic cue display was irrelevant to the task, whereas in the mixed condition each cue symbol signaled the type of saccade to be executed in the upcoming trial.

The sequence of each trial was as follows. A white fixation cross was displayed centrally on a black background for between 1,000 and 1,500 ms in 100-ms increments to ensure that participants were looking at the centre of the screen at the start of each trial, and to minimize head movements. The fixation cross was then replaced with a central cue (diamond or circle) for 1,000 ms. In the blocked condition, these symbolic cues had no significance, whereas in the mixed condition they signaled on a trial by trial basis whether a pro or an antisaccade should be made. The reason for having the cue display without any meaning in the blocked condition was to keep the display sequences constant across both conditions, to ensure that each condition had the same timings and, most importantly for this experiment, to enable us to manipulate the level of attention needed for each condition, which we did by making sure that participants had to focus on the cue on every trial in the mixed condition. The cue display was then replaced with a target display, where a single square target was presented for 1,000 ms randomly at one of four possible locations (2 directions: left or right, 2 eccentricities: near 2.5° or far 7°, from the centre of the display). Two target eccentricities were chosen to minimize the predictability of the target location, and hence reduce anticipatory saccades [34]. Cues and targets measured 1°×1° of visual angle, and all displays had black backgrounds with white stimuli. Figure 1 shows a schematic of the trial sequence.

### Design

The design was a 2 (age group: younger or older adults)×2 (saccade type: prosaccade or antisaccade)×2 (condition: blocked or mixed) factorial design with saccade type as the within-participant variable.

### Procedure

Participants were instructed to look toward the visually presented target as quickly and as accurately as possible in prosaccade trials, or to look at the mirror location opposite to the location of the visually presented target in antisaccade trials.

In the blocked task participants completed two prosaccade blocks and two antisaccade blocks, each of which consisted of 16 practice trials and 48 experimental trials. Blocks were counterbalanced in an ABAB-BABA design.

In the mixed task the centrally presented cue (diamond or circle) that changed randomly across trials, informed participants to direct their saccades either to the target or to the opposite location to the target. Each participant completed four blocks, each of which consisted of 16 practice trials and 48 experimental trials, and the symbol meaning was counterbalanced across participants.

Preceding each experimental block participants performed a calibration procedure where nine points in a square grid had to be fixated sequentially.

### Data Preparation

The display screen was divided into areas of interest to check whether participants’ saccadic landing positions were at the correct location. Exclusions included trials where the initial saccade fell outside the areas of interest (above or below), or where fixation at the moment of target onset was not within the central area of interest, or, if there were technical problems, blinks at trial onset or if the eye movement latencies were below 80 ms, as these were classified as anticipatory saccades, and in line with other papers any latencies that were greater than 1000 ms [9], [14]. A total of 7.8% (the blocked condition) and 6.2% (The mixed condition) of trials were excluded from analyses.

Correct responses were classified as trials, where the first saccade was located in the target area of interest in the prosaccade task (within 2 degrees of the edge of either target) or in the mirror area of interest in antisaccade task. Errors were classified as trials where the first saccade was executed in the direction opposite to the appropriate location for that trial.

## Results

We compared the eye movement data separately for proportions of errors, and latencies for correct responses in two repeated measures ANOVAs with 2 saccade type (prosaccade versus antisaccade) as a within participant variable, and 2 condition (blocked versus mixed) and 2 group (older versus younger) as between participant variables.

### Direction Error Rate

The ANOVA yielded two main effects. These were: saccade type (pro 2%, anti 37%, *F*(1,76) = 281.72, *p*<.0001), and group (younger 14%, older 25%, *F*(1,76) = 25.25, *p*<.0001). More errors were made in the antisaccade task compared to the prosaccade task, and more errors were made by the older, compared to the younger group. Table 1 shows the mean proportion of errors for all conditions and both groups.

**Table 1:** Error rate (%) and correct latency (ms) in each condition for both groups (means ± *SD*).

		Error rate		Correct latency	
		Prosaccade	Antisaccade	Prosaccade	Antisaccade
Blocked	Younger	1 (1)	30 (18)	150 (15)	281 (43)
	Older	3 (4)	46 (17)	183 (22)	307 (64)
Mixed	Younger	2 (2)	25 (21)	162 (16)	275 (39)
	Older	2 (4)	49 (19)	175 (20)	275 (52)

**Figure 2:**
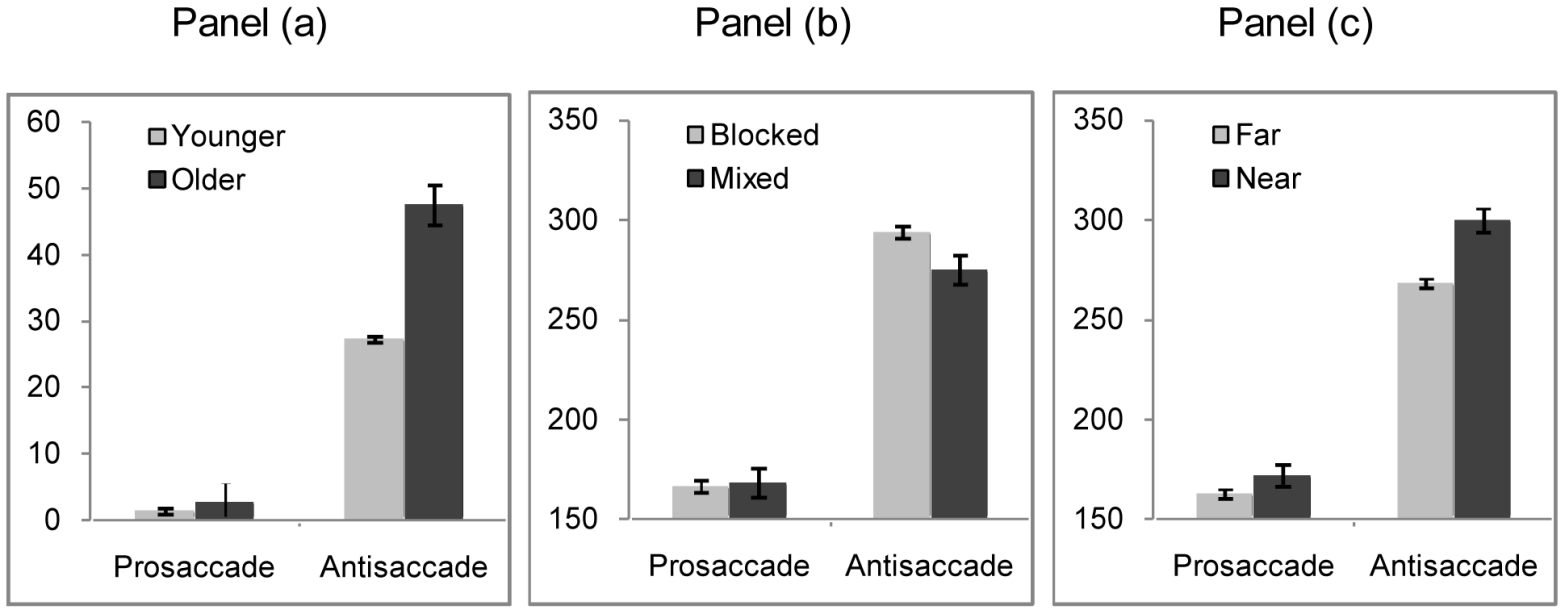
Error rate (%) and correct latency (ms) under different conditions. Panel (a). The mean direction error rate for prosaccades and antisaccades for younger and older participants. Panel (b). The mean correct latency for prosaccade and antisaccade tasks in the blocked condition and the mixed condition. Panel (c). The mean correct latency for near and far targets for younger and older participants. Error bars denote 1 standard error from the mean.

These main effects were qualified by a two-way interaction. This was between group and saccade type (*F*(1,76) = 20.03, *p*<.0001) and showed that older participants made significantly more errors in the antisaccade task than the younger participants (older antisaccade 48%, younger antisaccade 27%, *t*(78) = 4.84, *p*<.0001), but they did not differ in the prosaccade task (older prosaccade 3%, younger prosaccade 1%, *t*(78) = 1.59, *p = *.116), see panel (a) of Figure 2. There was no main effect of condition (*F*(1,76) = <1), and there was no 2 way interaction between condition and saccade type (*F*(1,76) *<*1), condition and group (*F*(1,76) <1), and no 3 way interaction between condition, saccade type and group (*F*(1,76)* = *1.76, *p = *.189). Older adults make more antisaccade errors for both conditions compared to the younger participants.

According to the goal neglect theory correct performance in conflict situations is dependent on adequate activation, or availability, of current task goals. If goal activation is insufficient when the stimulus is presented during the task a prepotent response will be executed instead. However, although the older participants make more errors in the antisaccade task compared to the younger participants in the current study, these findings cannot be a result of differences in the level of goal maintenance for the older group between the two conditions, since the younger group also showed the same pattern. Increasing goal activation in the mixed condition, by ensuring that attention to the central cue was necessary on all trials, did not reduce the proportion of errors in either participant group. The error data are more in line with inhibition theory than goal neglect theory since errors in the antisaccade task were uninfluenced by whether these were presented in blocked or mixed trials.

### Correct Trial Latencies

Latency was defined as the time elapsing from the onset of the target display to the initiation of correctly executed eye movements. Table 1 shows the mean correct latencies for all conditions and both groups. Main effects were observed for saccade type (prosaccade 167 ms, antisaccade 284 ms, *F*(1,76) = 661.08, *p*<.0001), and group (younger 217 ms, older 235 ms, *F*(1,76) = 7.97, *p*<.01). However, there was no interaction between saccade type and group (*F*(1,76) = 1.15, *p = *.287), which indicates that the commonly observed effect of longer latencies for older participants in antisaccade tasks, compared to younger participants [7], was not found here. An interaction between condition and saccade type (*F*(1,76) = 5.14, *p*<.05), see panel (b) in Figure 2, showed that all participants had marginally significantly longer saccade latencies in the antisaccade task in the blocked condition compared to mixed condition (blocked antisaccade 294 ms, mixed antisaccade 275 ms, *t*(78) = 1.92, *p = *.063), but they did not differ for prosaccades in the two conditions (blocked prosaccade 167 ms, mixed prosaccade 168 ms, *t*<1). What this means is that in the mixed condition, the latencies for the antisaccades are reduced compared to those made in the blocked condition. Moreover, since this effect did not interact with group (*F*(1,76) = 0.11, *p = *.747), this indicates that the improvements in performance for the antisaccades in the mixed condition compared to the blocked condition are observed in both the younger and the older adults in this study. This pattern of results does not support the goal neglect theory. If goal neglect was responsible for producing longer latencies in the older participants then we would have expected to see longer latencies in blocked compared to mixed conditions for the antisaccades for the older, but not the younger participants. We realize that the lack of a three way interaction could reflect a reduction in power for that test, compared to that needed for a two way interaction but we do not think that this is the case here, as a power analyses reveals that the number of participants that would be needed to ensure that a three-way interaction reached significance would be >1000.

### Switch Cost Analyses

In the mixed condition there is also a task switching component, which is absent in the blocked condition. It is therefore important to show that any differences in performance between the two conditions did not occur as a result of any task switching effects. In previous research older participants have shown evidence of task switching costs in several studies [35]–[37]. However, in the current study the reduced latencies in the mixed condition might be due to a switch benefit, rather than a cost [28]. In order to check that the latency decreases in the mixed condition were not a result of any switch benefit, we took the data from the mixed condition and separated the trials into repeat or switch trials [28]. Switch trials were categorized as such when the preceding trial type was different from the current one, and repeat trials were categorized as such if the preceding trial was the same as the current trial. We carried out a repeated measures ANOVA with 2 saccade type (prosaccade versus antisaccade) and 2 trial type (switch versus repeat) as within participant variables, and 2 group (older versus younger) as between participant variables for the saccade latencies and error proportions in the mixed condition. The results showed that the latencies did not differ for switch or repeat trials (there was no main effect of trial type (*F*(1,38) = 1.44, *p = *.310), and trial type did not come into any significant interaction, (all F’s<1). Therefore, because switching or repeating the same trial type throughout the task had no impact on the latencies for either group, we can be confident that the two-way interaction (saccade type by condition) we reported earlier in our latency analyses, was purely due to the increased attentional focus, rather than any switch costs (or benefits) in the mixed condition. Table 2 shows the mean latencies and proportion of errors for all conditions and both groups for the switch cost analysis.

**Table 2:** Error rate (%) and correct latency (ms) for switch and repeat trials in the mixed condition (means ± *SD*).

		Switch trials		Repeat trials	
		Prosaccade	Antisaccade	Prosaccade	Antisaccade
Latencies	Younger	165 (17)	272 (38)	159 (20)	277 (41)
	Older	175 (22)	277 (47)	173 (21)	273 (53)
Errors	Younger	2 (3)	25 (17)	2 (3)	25 (22)
	Older	2 (3)	49 (21)	2 (2)	49 (23)

### Eccentricity Effects

Eccentricity was used to minimize the proportion of anticipatory saccades in this experiment and was therefore not assumed to have any effect upon the dependent variables of errors and latencies across the two conditions or participant groups. We checked to see if this was the case and found main effects of increased latencies for correctly executed saccades for near compared to far targets (near 236 ms, far 215 ms, *F*(1,76) = 59.13, *p*<.0001) and increased error proportions for near compared to far targets (near 18.5%, far 20.3%, *F*(1,76) = 4.42, *p*<.05).

The main effect of latency showed that latencies were longer for near compared to far targets, and this finding was uninfluenced by whether participants were making pro or antisaccades. An interaction between eccentricity and saccade type (*F*(1,76) = 23.45, *p<*.0001) indicated that latencies were longer for near targets compared to far targets for both pro (*t*(79) = 5.78, *p*<.0001), and antisaccades (*t*(79) = 6.65, *p*<.0001) see panel (c) in Figure 2.There were no other main effects or interactions.

There is some evidence to show that it takes longer to initiate a saccade for near targets (2 degrees) for pro and antisaccade conditions in younger adults [38], and our findings suggest that this applies to older adults too. One possible explanation for this effect is that near targets produce activity in both saccade fixation and saccade initiation cells in the superior colliculus and the increase in time taken to initiate small amplitude saccades results from the time taken to resolve such conflict, the prolonged latencies resulting from heightened activity in the fixation area of the superior colliculus which needs to be suppressed in order to execute a saccade either to, or away from the near target [34].

## Discussion

In two experimental conditions we manipulated the focus of attention in an antisaccade task. This was achieved by presenting antisaccades in mixed (high attentional focus) or blocked (low attentional focus) trials. In the blocked condition participants were informed as to the nature of the goal at the beginning of each block whereas in a mixed condition the information as to whether to initiate a prosaccade, or an antisaccade was signaled on a trial-by-trial basis. As such we argue that the focus of attention in the mixed condition was higher.

According to the inhibition theory, poorer performance in older participants should result in the error rates for antisaccade trials being greater for the older participants in both a low and a high attentional focus condition (blocked and mixed), and this is what we found. Moreover, if inhibition accounts for observed deficits in older people the latencies for high and low attentional focus conditions should show the same pattern for younger and older participants, irrespective of differences in goal activation, and this was also observed in this study. However, both groups showed a decrease in antisaccade latencies for the mixed versus the blocked condition. Increasing goal activation in the mixed condition appears to increase the ability to inhibit distractor activation for the voluntary control system since both groups are able to initiate saccades to the opposite location of a target faster in that condition.

Older participants in this study were more efficient in the mixed trial condition compared to the blocked condition, but were equally ineffective in both conditions, showing more errors compared to the younger group and therefore poorer control over reflexive orienting. Increasing goal activation by modulating attentional demands of the task improved voluntary control in older group, but not involuntary control. It should be noted that the younger group’s error rates were also unaffected by increasing the attentional demands, and so in theory, effectiveness in both groups remained the same across both conditions. It could be argued that effectiveness is about competency and not about speed, it is about the ability to inhibit inappropriate prepotent responses, and therefore it should be unaffected by whether participants have to pay more or less attention to a central cue during the task.

Our findings for older and younger adults are consistent with the paradoxical effect of better performance in a mixed rather than a blocked antisaccade task [28]. However, we found no switch benefit in the mixed condition, in that the latencies did not differ for switch or repeat trials. This suggests that it is an increase in the focus of attention in the mixed block that reduces the time taken to initiate an antisaccade.

The paradoxical effect though, is not always observed [9]. This may be because in that study an overlap condition, where the central cue remained on throughout each trial, was adopted. It is known that this ‘overlap’ condition produces longer saccade latencies compared to when the central cue is offset either simultaneous with or before the target display appears. However, and in line with our findings reported in the current paper, the single notable group difference in the Bojko paper was of an increase in errors in the antisaccade trials for the older adults compared to the younger adults, across both conditions.

In another study, goal maintenance was kept the same whilst inhibition was modulated [12]. In one condition it was assumed that it would be more difficult to inhibit a prepotent response (to a flashing peripheral cue) than in another easier condition (to a static central cue), and older participants made more errors in both conditions, and showed longer latencies for the peripheral cue compared to the central cue for antisaccades. In our current study reported here we manipulated focused attention which should have increased goal activation for one condition over another, and yet still found that the older participants made more errors in both conditions.

If poorer performance in older participants is because of a failure to maintain the goal in an antisaccade task, then there should have been differences in performance between older and younger adults for a task with high goal maintenance compared to one with low goal maintenance. This is in agreement with Butler et al., (1999), who found that saccade latencies increased by the same amount in older adults and younger adults for the antisaccade task relative to the prosaccade task, whereas the proportion of directional errors in the antisaccade task was disproportionally higher for older adults [8]. Thus, although our data may not be able to provide an unequivocal resolution to the goal neglect versus the inhibition account of poor performance in older participants in the antisaccade task, they do provide more support for the view that it is the inhibition system, rather than goal neglect, which deteriorates with age.

The theory of attentional control may be also be relevant in understanding inhibition or goal neglect accounts of performance in the antisaccade task [39]. “ In *inhibition*, attentional control prevents attentional resources from being allocated to task irrelevant stimuli, and in *shifting* attentional control is used in a positive way to allocate attentional resources to execute the task relevant to the current goal” [28]. In attentional control theory a distinction is made between efficiency (the amount of processing resources invested) in completing the task, and effectiveness (competence) in completing the task. This means that if more attention is allocated to a mixed antisaccade condition compared to a blocked condition, there should be a reduction in latencies reflecting an increase in efficiency. However, increasing the processing resources should not impact upon errors, since effectiveness reflects an ability to inhibit a reflexive response to a stimulus onset.

We have found evidence of positive effects on efficiency, with both groups improving performance in the mixed versus the blocked trials with respect to latency for antisaccade. Additionally, we have also found that effectiveness, as measured by accuracy, was influenced by inhibition deficits in older participants. However, the ability to inhibit making reflexive responses was not affected by differences in attentional focus in the older participants.

In summary, we have found that older and younger people show a paradoxical effect of an improvement in performance for antisaccade latencies in a condition where attention has to be focused throughout the task. Additionally, age was a factor in the ability to inhibit reflexive responses, and this ability was not modulated by increasing attentional focus in our older group. Our data are best explained by the inhibitory deficit hypothesis, where inhibition, as defined by attentional control theory, can account for both the latency and error data in this study.
